# Impact of adjuvant chemotherapy for radically resected esophageal squamous cell carcinoma: a propensity score matching analysis

**DOI:** 10.3389/fsurg.2023.1181505

**Published:** 2023-05-03

**Authors:** Shao-bin Chen, Di-tian Liu, Yu-ping Chen

**Affiliations:** Department of Thoracic Surgery, Cancer Hospital of Shantou University Medical College, Shantou, China

**Keywords:** adjuvant chemotherapy, esophageal neoplasm, prognosis, squamous cell carcinoma, surgery

## Abstract

**Background:**

The aim of this study was to evaluate the impact of adjuvant chemotherapy in patients with radically resected esophageal squamous cell carcinoma (ESCC).

**Methods:**

Patients with esophageal cancer who underwent esophagectomy at our hospital from 2010 to 2019 were retrospectively analyzed. Only patients with radically resected ESCC who did not receive neoadjuvant therapy or adjuvant radiotherapy were enrolled in this study. Propensity score matching (1:1) was used to balance the baseline.

**Results:**

A total of 1,249 patients met the inclusion criteria and were enrolled in the study, and 263 patients received adjuvant chemotherapy. After matching, 260 pairs were analyzed. The 1-, 3-, and 5-year overall survival (OS) rates were 93.4%, 66.1% and 59.6%, respectively, for patients with adjuvant chemotherapy compared with 83.8%, 58.4% and 48.8%, respectively, for patients with surgery alone (*P* = 0.003). The 1-, 3-, and 5-year disease-free survival (DFS) rates were 82.3%, 58.8% and 51.3%, respectively, for patients with adjuvant chemotherapy compared with 68.0%, 48.3% and 40.8%, respectively, for patients with surgery alone (*P* = 0.002). In multivariate analyses, adjuvant chemotherapy was found to be an independent prognostic factor. In subgroup analyses, only the patients in certain subgroups were found to benefit from adjuvant chemotherapy, such as patients who underwent right thoracotomy, pT3 diseases, pN1-pN3 diseases, or pTNM stage III and IVA diseases.

**Conclusions:**

Postoperative adjuvant chemotherapy can improve the OS and DFS of ESCC patients after radical resection but may only work for patients in certain subgroups.

## Introduction

Esophageal cancer is the seventh commonly diagnosed cancer and sixth leading cause of cancer death in the world (1). An estimated 21,560 people were diagnosed with esophageal cancer, and 16,120 people were eventually died of their disease in the USA in 2023 (2). Esophageal cancer is one of the most common malignancies in China (3), and esophageal squamous cell carcinoma (ESCC) is the predominant histological type (4). Surgical resection is still a standard therapeutic approach for patients with resectable ESCC, but the prognosis is still disappointing (4). Although neoadjuvant chemoradiotherapy plus surgery is currently recommended for patients with locally advanced ESCC, it is still an infrequently used procedure in China. In a previous study that analyzed the national database of China, neoadjuvant therapy was only given to 18.5% of the patients with esophageal cancer in 2015 (6.2% of patients received neoadjuvant chemoradiotherapy, 9.0% of patients received neoadjuvant chemotherapy, and 3.3% of patients received neoadjuvant radiotherapy), while 21.2% of the patients received adjuvant chemotherapy (5).

The survival benefit of postoperative adjuvant chemotherapy has been demonstrated in many malignancies, including non-small cell lung carcinoma, gastric cancer, breast cancer, and colon cancer (6–9). However, the efficacy of adjuvant chemotherapy on ESCC is still controversial. Few randomized, controlled trials have been conducted to explore the efficacy of adjuvant chemotherapy in ESCC patients after radical surgery due to disappointing results (10, 11). Currently, no optimal postoperative adjuvant therapy is recommended in the National Comprehensive Cancer Network (NCCN) guidelines. However, an increasing number of retrospective studies have found that adjuvant chemotherapy could significantly improve survival in ESCC patients after radical resection (12–15).

Therefore, we think that the role of adjuvant chemotherapy in patients with ESCC should be further elucidated. In this study, we retrospectively assessed the efficacy of adjuvant chemotherapy in ESCC patients after radical resection compared with those who underwent surgery alone. Propensity score matching (PSM) was also used in this study to minimize baseline differences between groups.

## Patients and methods

### Patients

A total of 2,324 patients with esophageal carcinoma underwent esophagectomy at Shantou University Medical College Cancer Hospital between May 2010 and July 2019. The inclusion criteria for this study were as follows: (1) thoracic esophageal squamous cell carcinoma; (2) no neoadjuvant therapy before surgery; (3) underwent complete resection (R0 resection); and (4) no adjuvant radiotherapy. Patients who met the following criteria were excluded: (1) cervical ESCC; (2) underwent incomplete resection (R1 or R2 resection); and (3) concurrent or previous history of other malignancies. Patients who survived less than 3 months or had tumor relapse within 3 months after esophagectomy were also excluded to remove possible bias in favor of the adjuvant chemotherapy group, as some of the patients who underwent surgery alone might have died or had tumor relapse before receiving adjuvant chemotherapy. Approval was obtained from the institutional review board, and informed consent was acquired from all participants.

Chest radiograph, barium meal, Doppler ultrasound examination of the supraclavicular lymph nodes, and contrast enhanced computed tomography scan of the chest and abdomen were routinely conducted to stage all patients before surgery. Endoscopic ultrsonography (EUS) was also performed for most of the patients after the year 2010. Positron emission tomography (PET) was not routinely performed before surgery.

### Surgery

Esophagectomy was performed through a right thoracotomy or left thoracotomy, and esophagogastric anastomosis was performed in a neck incision for most of the patients. The thoracotomy was usually performed on the left for tumors located below the aortic arch and on the right for tumors located above the aortic arch before 2010, however, a right thoracotomy was routinely performed for most of the patients after 2011. Minimally invasive esophagectomy (MIE) was also performed after 2011. In all patients, a standard abdominal lymphadenectomy (left and right paracardial regions, along the lesser curve and the left gastric artery) and mediastinal lymphadenectomy (subcarinal, left and right bronchial, lower posterior mediastinum, pulmonary ligament, paraesophageal and thoracic duct) were performed. For patient who underwent a right thoracotomy or MIE, the common hepatic nodes, left and right recurrent laryngeal nerve lymph nodes were also dissected. Pathological stage was defined based on the eighth edition TNM classification.

### Chemotherapy

Chemotherapy was first administered to patients at 4–8 weeks after the surgery. The most commonly used chemotherapy included the 5-fluorouracil plus cisplatin regimen, docetaxel plus cisplatin regimen, docetaxel plus nedaplatin regimen, and S-1 single-agent (tegafur, gimeracil, and oteracil potassium capsules). Combination chemotherapy was administered every 3–4 weeks for 1–6 cycles (median 4 cycles). S-1 (80–120 mg/day, d1–14, q3w) single-agent chemotherapy was administered every 3 weeks for 1 year or until tumor recurrence.

### Statistical analyses

Pearson's *χ*^2^ test or Fisher's exact test was used to compare categorical variables. The Kaplan–Meier method was used to compare overall survival (OS) and disease-free survival (DFS) between groups, and the log-rank test was used to test the survival differences. Variables with *P* < 0.2 in univariate analysis were included in multivariate Cox regression analysis to investigate independent prognostic factors. PSM was performed with the 1:1 nearest neighbor matching method and included the following covariates: sex, age, tumor location, tumor length, histologic grade, body mass index (BMI), thoracotomy, pT category, pN category, and pTNM stage. *P* < 0.05 was considered statistically significant. All statistical analyses were conducted using SPSS 26.0 software (IBM, Armonk, New York, USA).

## Results

### Patient characteristics

**Figure 1:**
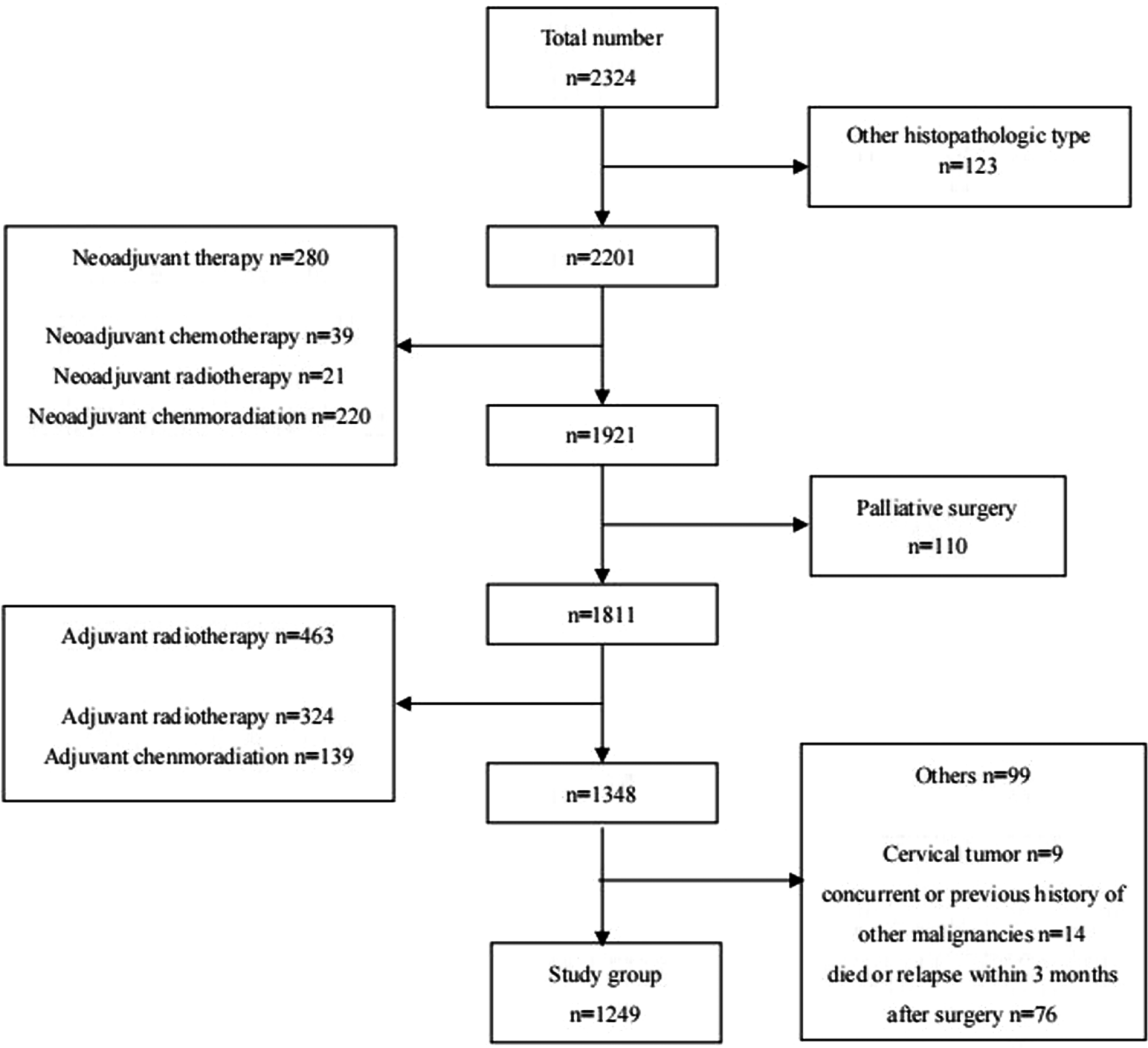
Patients with esophageal cancer underwent surgical resection at shantou university medical college cancer hospital between May 2010 and July 2019.

A total of 1,249 patients were enrolled in this study (Figure 1). The clinicopathological features of the study group are shown in Table 1. The median age for the whole group was 61 years (range, 37–84 years). Two hundred and seventy-five patients were underweight (BMI < 18.5 kg/m^2^), and 974 patients were normal weight or obese (BMI ≥ 18.5 kg/m^2^). The mean number of LNs removed was 26.2 ± 11.2, and the median number was 25 (range, 3–85). LN metastases were found in 529 patients (42.4%).

**Table 1:** Clinicopathological characteristics of the patients before and after propensity score matching.

Variable	Original cohort		*P*-value	Matched cohort		*P*-value
	S group (*n* = 986)	S + C group (*n* = 263)		S group (*n* = 260)	S + C group (*n* = 260)	
Sex			0.002			0.912
Male	697 (70.7%)	211 (80.2%)		208 (80.0%)	209 (80.4%)	
Female	289 (29.3%)	52 (19.8%)		52 (20.0%)	51 (19.6%)	
Age (year)			0.094			0.332
≤60	467 (47.4%)	150 (53.0%)		137 (52.7%)	148 (56.9%)	
>60	519 (52.6%)	133 (47.0%)		123 (47.3%)	112 (43.1%)	
Tumor location			0.050			0.506
Upper third	135 (13.7%)	26 (9.9%)		25 (9.6%)	25 (9.6%)	
Middle third	665 (67.4%)	172 (65.4%)		181 (69.6%)	170 (65.4%)	
Lower third	186 (18.9%)	65 (24.7%)		54 (20.8%)	65 (25.0%)	
Tumor length			<0.001			0.643
≤4 cm	530 (53.8%)	86 (32.7%)		90 (34.6%)	85 (32.7%)	
>4 cm	456 (46.2%)	177 (67.3%)		170 (65.4%)	175 (67.3%)	
Thoracotomy			0.006			0.617
Left thoracotomy	352 (35.7%)	70 (26.6%)		65 (25.0%)	70 (26.9%)	
Right thoracotomy	634 (64.3%)	193 (73.4%)		195 (75.0%)	190 (73.1%)	
BMI (kg/m^2^)			0.247			0.661
<18.5	224 (22.7%)	51 (19.4%)		54 (20.8%)	50 (19.2%)	
≥18.5	762 (77.3%)	212 (80.6%)		206 (79.2%)	210 (80.8%)	
Histologic grade			0.009			0.757
Well	352 (35.7%)	73 (27.8%)		70 (26.9%)	73 (28.1%)	
Moderate	491 (49.8%)	135 (51.3%)		130 (50.0%)	134 (51.5%)	
Poor	143 (14.5%)	55 (20.9%)		60 (23.1%)	53 (20.4%)	
pT category			<0.001			0.426
pT1	200 (20.3%)	14 (5.3%)		9 (3.5%)	14 (5.4%)	
pT2	198 (20.1%)	30 (11.4%)		40 (15.4%)	30 (11.5%)	
pT3	572 (58.0%)	210 (79.8%)		204 (78.5%)	207 (79.6%)	
pT4	16 (1.6%)	9 (3.4%)		7 (2.7%)	9 (3.5%)	
pN category			<0.001			0.544
pN0	679 (68.9%)	41 (15.6%)		53 (20.4%)	41 (15.8%)	
pN1	184 (18.7%)	115 (43.7%)		106 (40.8%)	115 (44.2%)	
pN2	91 (9.2%)	77 (29.3%)		70 (26.9%)	75 (28.8%)	
pN3	32 (3.2%)	30 (11.4%)		31 (11.9%)	29 (11.2%)	
pTNM stage			<0.001			0.936
I	255 (25.9%)	12 (4.6%)		11 (4.2%)	12 (4.6%)	
II	440 (44.6%)	41 (15.6%)		46 (17.7%)	41 (15.8%)	
III	257 (26.4%)	177 (67.3%)		170(65.4%)	175(67.3%)	
IVA	34 (3.4%)	33 (12.5%)		33(12.7%)	32(12.3%)	

BMI, body mass index; C, chemotherapy; S, surgery.

Two hundred and sixty-three patients received adjuvant chemotherapy (S + C group), including 52 patients with 5-fluorouracil plus cisplatin chemotherapy, 95 patients with docetaxel plus cisplatin chemotherapy, 67 patients with docetaxel plus nedaplatin chemotherapy, and 49 patients with S-1 single-agent chemotherapy. Compared with patients who underwent surgery alone (S group), there were more males (*P* = 0.002) with longer tumor lengths (*P* < 0.001) in the S + C group. Moreover, patients in the S + C group underwent more right thoracotomy (*P* = 0.006) and had higher histological grade (*P* = 0.009) and advanced-stage tumors (*P* < 0.001). After 1:1 PSM, 260 well-balanced pairs were enrolled for further analysis (Table 1).

### Survival and prognostic factors

The follow-up data were updated to June 2022, and the mean follow-up time was 62.1 months (range, 4–145 months). In the whole study group of 1,249 patients, 540 patients had recurrent diseases, 468 patients had died, and 33 patients were lost to follow-up (2.6%). The 1-, 3- and 5-year OS rates for the entire study group were 92.1%, 72.6% and 64.7%, respectively, and the 1-, 3- and 5-year DFS rates were 82.1%, 64.9% and 58.2%, respectively.

**Figure 2:**
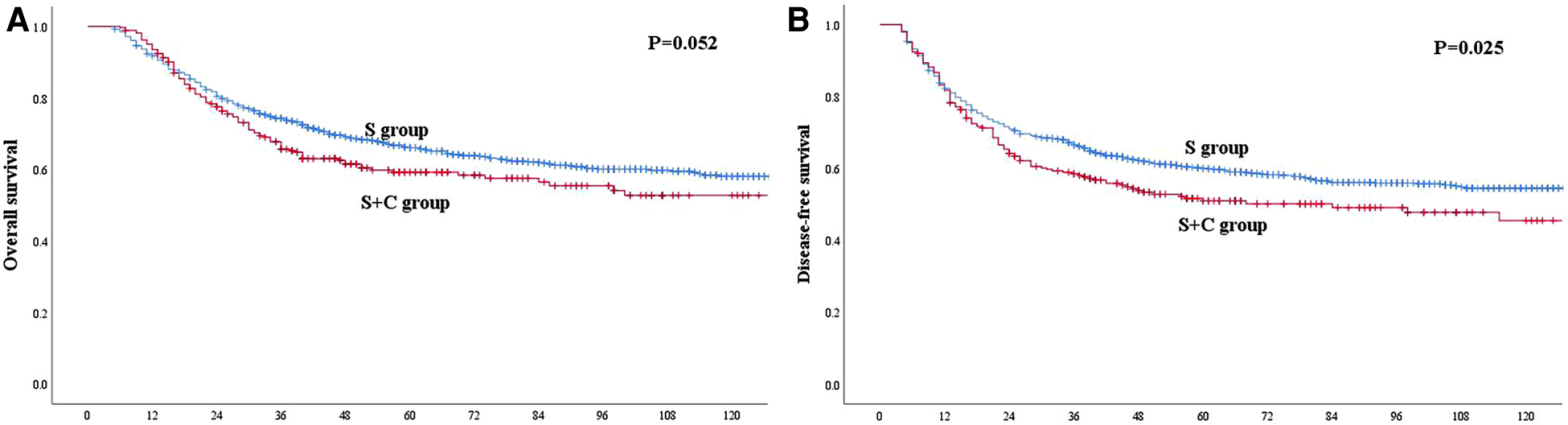
(**A**) Kaplan–Meier curves for overall survival of the patients in the whole study group. The survival was better for patients in S group than patients in S + C group. However, the difference was not significant (*P* = 0.052). (**B**) Kaplan–Meier curves for disease-free survival of the patients in the whole study group. The survival was significantly better for patients in S group than patients in S + C group (*P* = 0.025).

**Figure 3:**
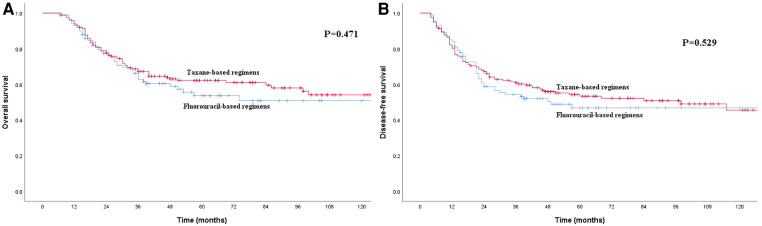
(**A**) Kaplan–Meier curves for overall survival of the patients received adjuvant chemotherapy. The survival difference between patients received taxane-based regimens chemotherapy and patients received fluorouraci-based regimens chemotherapy was not significant (*P* = 0.471). (**B**) Kaplan–Meier curves for disease-free survival of the patients received adjuvant chemotherapy. The survival difference between patients received taxane-based regimens chemotherapy and patients received fluorouraci-based regimens chemotherapy was not significant (*P* = 0.529).

Before PSM, the 1-, 3- and 5-year OS rates for patients in the S group were 91.8%, 74.3% and 66.1%, respectively, which were better than the rates of 93.5%, 65.7% and 59.2%, respectively, among patients in the S + C group (Figure 2A), although the *P*-value of 0.052 indicated that the difference was nonsignificant. The 1-, 3- and 5-year DFS rates for patients in the S group were 82.2%, 66.6% and 60.0%, respectively, which were higher than those of 81.7%, 58.5% and 51.0%, respectively, for patients in the S + C group (*P* = 0.025, Figure 2B). The 5-year OS rate for patients received taxane-based regimens chemotherapy (docetaxel plus cisplatin or docetaxel plus nedaplatin) was 62.2%, compared with that of 53.7% for patients received fluorouraci-based regimens chemotherapy (5-fluorouracil plus cisplatin or S-1 single-agent) (*P* = 0.471, Figure 3A). The 5-year DFS rate for patients received taxane-based regimens chemotherapy was 53.4%, compared with that of 46.8% for patients received fluorouraci-based regimens chemotherapy (*P* = 0.529, Figure 3B).

**Figure 4:**
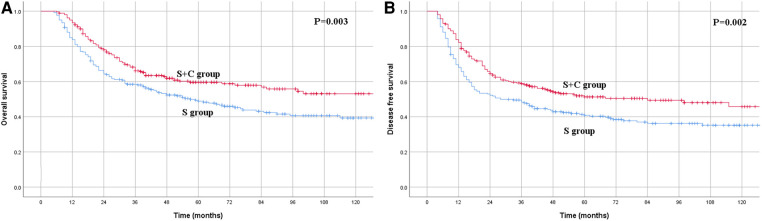
(**A**) Kaplan–Meier curves for overall survival of the patients in the propensity score matching group. The survival was significantly better for patients in S + C group than patients in S group (*P* = 0.003). (**B**) Kaplan–Meier curves for disease-free survival of the patients in the propensity score matching group. The survival was significantly better for patients in S + C group than patients in S group (*P* = 0.002).

In the PSM cohort, the 1-, 3- and 5-year OS rates for patients in the S group were 83.8%, 58.4% and 48.8%, respectively, compared with the rates of 93.4%, 66.1% and 59.6%, respectively, for patients in the S + C group (Figure 4A), and the difference was significant (*P* = 0.003). The 1-, 3- and 5-year DFS rates for patients in the S group of 68.0%, 48.3% and 40.8%, respectively, were also significantly worse than those of 82.3%, 58.8% and 51.3%, respectively, for patients in the S + C group (*P* = 0.002, Figure 4B). Other factors that were significantly correlated with survival included tumor length, pN category, and pTNM stage (Table 2).

**Table 2:** Univariate analysis in regard to overall survival and disease-free survival according to clinicopathological factors for the matched cohort.

Variable	5-years OS (%)	*P*-value	5-years DFS (%)	*P*-value
Sex		0.602		0.836
Male	54.5		46.0	
Female	52.8		46.1	
Age (year)		0.583		0.423
≤60	55.1		45.1	
>60	52.4		46.6	
Tumor location		0.095		0.500
Upper third	66.7		54.4	
Middle third	52.4		44.6	
Lower third	53.5		46.9	
Tumor length		**0** **.** **008**		**0**.**048**
≤4 cm	61.5		50.7	
>4 cm	50.4		43.6	
Thoracotomy		0.173		0.178
Left thoracotomy	50.0		41.1	
Right thoracotomy	55.8		48.0	
BMI (kg/m^2^)		0.296		0.321
<18.5	51.5		43.6	
≥18.5	55.1		46.9	
Histologic grade		0.696		0.574
Well	56.3		46.7	
Moderately	53.9		47.2	
Poorly	52.8		41.7	
pT category		0.051		0.066
pT1	76.1		64.5	
pT2	56.4		52.1	
pT3	53.1		44.3	
pT4	37.0		36.5	
pN category		**<0**.**001**	** **	**<0**.**001**
pN0	81.7		72.7	
pN1	60.2		53.6	
pN2	37.3		31.3	
pN3	28.4		12.4	
pTNM stage		**<0**.**001**	** **	**<0**.**001**
I	76.1		59.3	
II	80.1		73.2	
III	50.8		44.4	
IVA	28.1		13.0	
Adjuvant chemotherapy		**0**.**003**	** **	**0**.**002**
No	48.8		40.8	
Yes	59.6		51.3	

BMI, body mass index; DFS, disease-free survival; OS, overall survival. The bold values indices are statistically significant.

In multivariate analysis, pN category and adjuvant chemotherapy were independently correlated with OS and DFS, while thoracotomy was only independently correlated with DFS. None of the other factors were independent risk factors in this matched cohort (Table 3).

**Table 3:** Multivariate analysis in regard to overall survival and disease-free survival of the patients in the matched cohort.

Prognostic factor	Hazard ratio	95% CI	*P*-value
Overall survival			
Tumor location	1.152	0.907–1.464	0.245
Tumor length	1.173	0.873–1.575	0.290
Thoracotomy	0.775	0.585–1.028	0.077
pT category	1.016	0.733–1.407	0.924
pN category	1.660	1.286–2.142	**<0** **.** **001**
pTNM stage	1.164	0.778–1.742	0.461
Adjuvant chemotherapy	0.579	0.448–0.748	**<0**.**001**
Disease-free survival			
Tumor length	1.021	0.781–1.336	0.877
Thoracotomy	0.769	0.593–0.997	**0**.**048**
pT category	1.101	0.816–1.486	0.528
pN category	1.856	1.469–2.346	**<0**.**001**
pTNM stage	1.093	0.756–1.579	0.636
Adjuvant chemotherapy	0.576	0.454–0.731	**<0**.**001**

CI, confidence interval. The bold values indices are statistically significant.

### Subgroup analyses for the impact of adjuvant chemotherapy

The impact of adjuvant chemotherapy on survival in subgroup analyses is shown in Table 4. Adjuvant chemotherapy was found to improve survival in patients who underwent right thoracotomy (*P* = 0.006) but not in patients who underwent left thoracotomy (*P* = 0.178). Patients with pT3 diseases, pN1-pN3 diseases, or pTNM stage III and IVA diseases were more likely to benefit from adjuvant chemotherapy than patients with pT1–2 diseases, pT4 diseases, pN0 diseases, or pTNM stage I–II diseases. Moreover, adjuvant chemotherapy was also found to improve survival in the subgroups of male patients, age >60 years, tumor >4 cm, tumor located in middle or lower third of the thorax, and moderately or poorly differentiated tumors.

**Table 4:** Impact of adjuvant chemotherapy on overall survival and disease-free survival in subgroup analyses.

Variable	No of patients	5-years OS (%)		*P*-value	5-years DFS (%)		*P*-value
		S	S + C		S	S + C	
Sex							
Male	417	49.2	60.0	0.009	40.1	52.0	0.003
Female	103	47.8	57.9	0.137	44.1	48.1	0.285
Age (year)							
≤60	285	53.9	56.2	0.445	42.4	47.5	0.167
>60	235	42.7	64.5	<0.001	38.5	56.1	0.001
Tumor location							
Upper third	50	61.5	70.4	0.373	43.6	66.0	0.053
Middle third	351	48.4	56.9	0.036	41.2	48.4	0.054
Lower third	119	43.3	62.0	0.036	38.9	53.2	0.034
Tumor length							
≤4 cm	175	59.0	64.1	0.212	46.9	55.2	0.228
>4 cm	345	43.4	57.4	0.004	37.4	49.4	0.002
Thoracotomy							
Left thoracotomy	135	46.0	53.8	0.178	39.7	42.3	0.446
Right thoracotomy	385	49.7	62.7	0.006	41.1	55.3	0.001
BMI (kg/m^2^)							
<18.5	104	44.0	58.8	0.106	37.2	50.0	0.125
≥18.5	416	50.4	59.8	0.071	42.5	51.2	0.069
Histologic grade							
Well	143	57.8	55.1	0.987	46.6	46,3	0.749
Moderately	264	47.0	61.0	0.007	40.5	53.8	0.009
Poorly	113	43.5	63.6	0.038	33.4	51.0	0.017
pT category							
pT1	23	88.9	64.3	0.547	88.9	47.6	0.158
pT2	70	54.4	59.4	0.416	49.1	56.3	0.434
pT3	411	46.7	59.7	0.004	37.8	50.8	0.001
pT4	16	17.1	51.9	0.276	14.3	53.3	0.080
pN category							
pN0	94	88.2	72.7	0.473	77.9	65.5	0.310
pN1	221	52.8	67.7	0.035	47.7	59.3	0.031
pN2	145	24.4	49.5	0.002	19.6	42.9	0.001
pN3	60	20.2	37.9	0.011	3.2	21.9	<0.001
pTNM stage							
I	23	90.9	59.5	0.489	79.5	41.7	0.104
II	87	86.4	72.1	0.458	77.5	68.1	0.497
III	345	41.3	60.6	0.001	35.8	53.1	<0.001
IVA	65	18.4	38.3	0.005	3.0	23.1	<0.001

BMI, body mass index; C, chemotherapy; DFS, disease-free survival; OS, overall survival; S, surgery.

## Discussion

The CROSS study published in 2012 established neoadjuvant chemoradiotherapy plus surgery as the standard treatment for locally advanced esophageal cancer (16). However, the long-term survival is still very disappointing, with a 10-year OS of only 38% for patients treated with the CROSS strategy (17). Recently, the CheckMate 577 trial showed that adjuvant nivolumab therapy could improve DFS for patients with residual disease after neoadjuvant chemoradiotherapy plus surgery (18). However, because of the different tumor prevalence and surgery preferences, the optimal treatment strategies for locally advanced ESCC remain unclear (19).

The efficacy of adjuvant chemotherapy on ESCC is still controversial. Few randomized trials have evaluated the effect of adjuvant chemotherapy on patients with ESCC after radical surgery. The JCOG8806 study revealed that no survival benefit was obtained from adjuvant chemotherapy using a combination of cisplatin and vindesine (10). In the JCOG9204 study, the 5-year DFS was significantly better when patients with positive LNs received adjuvant chemotherapy with cisplatin plus fluorouracil (52.0% vs. 38.0%; *P* = 0.041); however, the difference for OS was not significant (11). Both of these clinical trials were conducted in an early period. Recently, newer agents such as the taxane-based regimens (docetaxel or paclitaxel), which are recognized may be more effective than typically used agent such as fluorouracil, have been used in adjuvant chemotherapy in patients with ESCC (12). Some recent retrospective studies and meta-analyses found that adjuvant chemotherapy could improve survival in certain subgroups for patients with ESCC after radical resection, especially for patients with positive LNs (12, 13, 15, 20, 21), indicating that the efficacy of adjuvant chemotherapy should be further determined.

Our current retrospective study enrolled one of the largest patient cohorts to date. We also used PSM to minimize baseline differences between the S group and the S + C group. Our results confirmed that adjuvant chemotherapy not only improved DFS for patients with ESCC who underwent radical resection but also improved OS, and adjuvant chemotherapy was an independent prognostic factor. These results were similar to some of the other retrospective studies (12, 13, 20). In a recent meta-analysis by Zhao et al. (21) that enrolled 9 studies and a total of 1,684 cases, the authors also found that adjuvant chemotherapy could improve OS (HR: 0.78, *P* = 0.002) and DFS (HR: 0.72, *P* < 0.001) for patients with ESCC. There may be two reasons for the positive results in our study. First, all of the surgeries were performed or closely supervised by two senior surgeons (J. S. Yang and Y. P. Chen). The homogeneity of the surgical treatment will reduce the methodological biases. Second, 88.2% of the patients in our study received more than 3 cycles of chemotherapy, and the median number was 4, while both of the randomized trials of adjuvant chemotherapy on ESCC used only 2 cycles (10, 11). Previous study also demonstrated that the effects of adjuvant chemotherapy were associated with the chemotherapy cycles (22).

Subgroup analyses in our study showed that not all patients benefited from adjuvant chemotherapy. Our findings that patients with pN1-pN3 diseases and pTNM stage III–IVA diseases, but not pN0 diseases and pTNM stage I–II diseases, were more likely to benefit from adjuvant chemotherapy were consistent with previous studies (15, 23). Patients with positive LNs and advanced TNM stage are known to have a high risk of tumor recurrence and should be more likely to have systemic disease, so systemic chemotherapy might improve the survival of these patients (14). Accordingly, patients with moderately and poorly differentiated tumors and tumor lengths >4 cm were also found to benefit from adjuvant chemotherapy, as previous studies showed that these patients might have a higher rate of tumor recurrence and worse survival (24, 25). However, Pasquer et al. (26) found that adjuvant chemotherapy did not improve the OS and DFS for esophageal cancer patients with positive LNs. Ando et al. (10) also found that adjuvant chemotherapy did not improve 5-year survival for LN-positive ESCC patients (43.7% vs. 35.5%, *P* = 0.13). The differences in surgical approach, lymphadenectomy, chemotherapy agents, and chemotherapy cycles might contribute to these different results.

Our subgroup analyses also showed that patients who underwent right thoracotomy were more likely to benefit from adjuvant chemotherapy than patients who underwent left thoracotomy. Bilateral recurrent laryngeal nerve LNs, with nearly 40% involvement, were the most frequent metastatic nodes in thoracic ESCC (27, 28). However, these LNs could not be removed through a left thoracotomy. This means that nearly 40% of ESCC patients who undergo left thoracotomy may not receive radical surgery, resulting in a high rate of locoregional recurrence. For these patients, postoperative chemoradiotherapy but not chemotherapy may be a better adjuvant therapy to reduce locoregional recurrence and improve survival (29, 30).

Surprisingly, we found that younger patients (≤60 years) obtained fewer survival benefits from adjuvant chemotherapy than older patients (>60 years) in our subgroup analyses, which was different from the result by Zhu et al. (23) Most previous studies have shown that age is a factor that influences treatment choices but not necessarily outcomes, and both younger patients and older patients could benefit comparably from chemotherapy (31, 32). We quite agree with this opinion. In fact, the 5-year OS and DFS for younger patients in S + C group were higher than those for younger patients in S group in our study, although the differences were not significant (*P* > 0.05). The reasons for fewer survival benefits obtained from adjuvant chemotherapy in younger patients may be that there are more patients with pN0 diseases in younger group than in older group (20% vs. 15.7%). According to our results, we think that adjuvant chemotherapy should not be withheld based on age alone in ESCC patients after surgery. Although we also found that adjuvant chemotherapy might not benefit female patients or patients with tumors located in the upper third of the thorax, the sample sizes in these subgroups were too small to draw a conclusion. We think that more data should be collected to evaluate these results.

There are some limitations to this study. First, it was a retrospective study, and we could not analyze the toxicity of adjuvant chemotherapy in this study, as most of these data were missing. Second, different chemotherapy agents, such as 5-fluorouracil, cisplatin, and docetaxel, were used, and we could not define the optimal chemotherapy regimens in this study. Third, although PSM was used to balance the baseline differences, some of the other factors that may impact the prognosis, such as performance status, were not included in this study. With the development of new drugs, such as taxanes, increasing data have shown that adjuvant chemotherapy may improve survival in patients with ESCC. We think that a multicenter, randomized clinical trial should be conducted to explore the role of adjuvant chemotherapy in patients with ESCC after radical surgery.

In conclusion, postoperative adjuvant chemotherapy improves the OS and DFS of ESCC patients after radical resection but may only work for patients in certain subgroups. Further multicenter, randomized clinical trials should be conducted to evaluate our findings.

## Data Availability

The original contributions presented in the study are included in the article/**Supplementary Material**, further inquiries can be directed to the corresponding author.

## References

[1] Sung H Ferlay J Siegel RL Laversanne M Soerjomataram I Jemal A Global cancer statistics 2020: GLOBOCAN estimates of incidence and mortality worldwide for 36 cancers in 185 countries. CA Cancer J Clin. (2021) 71(3):209–49. 10.3322/caac.21660 33538338

[2] Siegel RL Miller KD Wagle NS Jemal A. Cancer statistics, 2023. CA Cancer J Clin. (2023) 73(1):17–48. 10.3322/caac.21763 36633525

[3] Chen W Zheng R Baade PD Zhang S Zeng H Bray F Cancer statistics in China, 2015. CA Cancer J Clin. (2016) 66(2):115–32. 10.3322/caac.21338 26808342

[4] Zeng H Zheng R Zhang S Zuo T Xia C Zou X Esophageal cancer statistics in China, 2011: estimates based on 177 cancer registries. Thorac Cancer. (2016) 7(2):232–7. 10.1111/1759-7714.12322 27042227 PMC4773307

[5] Qiu ML Lin JB Li X Luo RG Liu B Lin JW. Current state of esophageal cancer surgery in China: a national database analysis. BMC Cancer. (2019) 19(1):1064. 10.1186/s12885-019-6191-2 31703631 PMC6839071

[6] Pignon JP Tribodet H Scagliotti GV Douillard JY Shepherd FA Stephens RJ Lung adjuvant cisplatin evaluation: a pooled analysis by the LACE collaborative group. J Clin Oncol. (2008) 26(21):3552–9. 10.1200/JCO.2007.13.9030 18506026

[7] Noh SH Park SR Yang HK Chung HC Chung IJ Kim SW Adjuvant capecitabine plus oxaliplatin for gastric cancer after D2 gastrectomy (CLASSIC): 5-year follow-up of an open-label, randomised phase 3 trial. Lancet Oncol. (2014) 15(12):1389–96. 10.1016/S1470-2045(14)70473-5 25439693

[8] Early Breast Cancer Trialists’ Collaborative Group (EBCTCG). Effects of chemotherapy and hormonal therapy for early breast cancer on recurrence and 15-year survival: an overview of the randomised trials. Lancet. (2005) 365(9472):1687–717. 10.1016/S0140-6736(05)66544-0 15894097

[9] André T Boni C Navarro M Tabernero J Hickish T Topham C Improved overall survival with oxaliplatin, fluorouracil, and leucovorin as adjuvant treatment in stage II or III colon cancer in the MOSAIC trial. J Clin Oncol. (2009) 27(19):3109–16. 10.1200/JCO.2008.20.6771 19451431

[10] Ando N Iizuka T Kakegawa T Isono K Watanabe H Ide H A randomized trial of surgery with and without chemotherapy for localized squamous carcinoma of the thoracic esophagus: the Japan clinical oncology group study. J Thorac Cardiovasc Surg. (1997) 114(2):205–9. 10.1016/S0022-5223(97)70146-6 9270637

[11] Ando N Iizuka T Ide H Ishida K Shinoda M Nishimaki T Surgery plus chemotherapy compared with surgery alone for localized squamous cell carcinoma of the thoracic esophagus: a Japan clinical oncology group study–JCOG9204. J Clin Oncol. (2003) 21(24):4592–6. 10.1200/JCO.2003.12.095 14673047

[12] Qin RQ Wen YS Wang WP Xi KX Yu XY Zhang LJ. The role of postoperative adjuvant chemotherapy for lymph node-positive esophageal squamous cell carcinoma: a propensity score matching analysis. Med Oncol. (2016) 33(4):31. 10.1007/s12032-016-0746-8 26922662

[13] Lyu X Huang J Mao Y Liu Y Feng Q Shao K Adjuvant chemotherapy after esophagectomy: is there a role in the treatment of the lymph node positive thoracic esophageal squamous cell carcinoma? J Surg Oncol. (2014) 110(7):864–8. 10.1002/jso.23716 24976079

[14] Heroor A Fujita H Sueyoshi S Tanaka T Toh U Mine T Adjuvant chemotherapy after radical resection of squamous cell carcinoma in the thoracic esophagus: who benefits? A retrospective study. Dig Surg. (2003) 20(3):229–35. 10.1159/000070390 12759503

[15] Zhang SS Yang H Xie X Luo KJ Wen J Bella AE Adjuvant chemotherapy versus surgery alone for esophageal squamous cell carcinoma: a meta-analysis of randomized controlled trials and nonrandomized studies. Dis Esophagus. (2014) 27(6):574–84. 10.1111/dote.12073 23621119

[16] van Hagen P Hulshof MC van Lanschot JJ Steyerberg EW van Berge Henegouwen MI Wijnhoven BP Preoperative chemoradiotherapy for esophageal or junctional cancer. N Engl J Med. (2012) 366(22):2074–84. 10.1056/NEJMoa1112088 22646630

[17] Eyck BM van Lanschot JJB Hulshof MCCM van der Wilk BJ Shapiro J van Hagen P Ten-Year outcome of neoadjuvant chemoradiotherapy plus surgery for esophageal cancer: the randomized controlled CROSS trial. J Clin Oncol. (2021) 39(18):1995–2004. 10.1200/JCO.20.03614 33891478

[18] Kelly RJ Ajani JA Kuzdzal J Zander T Van Cutsem E Piessen G Adjuvant nivolumab in resected esophageal or gastroesophageal junction cancer. N Engl J Med. (2021) 384(13):1191–203. 10.1056/NEJMoa2032125 33789008

[19] Li B Chen H. The best surgery should be applied for locally advanced esophageal cancer. J Clin Oncol. (2021) 39(28):3189–90. 10.1200/JCO.21.01340 34339257

[20] Hashiguchi T Nasu M Hashimoto T Kuniyasu T Inoue H Sakai N Docetaxel, cisplatin and 5-fluorouracil adjuvant chemotherapy following three-field lymph node dissection for stage II/III N1, 2 esophageal cancer. Mol Clin Oncol. (2014) 2(5):719–24. 10.3892/mco.2014.320 25054036 PMC4106741

[21] Zhao P Yan W Fu H Lin Y Chen KN. Efficacy of postoperative adjuvant chemotherapy for esophageal squamous cell carcinoma: a meta-analysis. Thorac Cancer. (2018) 9(8):1048–55. 10.1111/1759-7714.12787 29927075 PMC6068451

[22] Duan J Deng T Ying G Huang D Zhang H Zhou L Prognostic nomogram for previously untreated patients with esophageal squamous cell carcinoma after esophagectomy followed by adjuvant chemotherapy. Jpn J Clin Oncol. (2016) 46(4):336–43. 10.1093/jjco/hyv206 26819278 PMC4886130

[23] Zhu K Ren P Yang Y Wang Y Xiao W Zhang H Role of chemotherapy after curative esophagectomy in squamous cell carcinoma of the thoracic esophagus: a propensity score-matched analysis. Thorac Cancer. (2021) 12(12):1800–9. 10.1111/1759-7714.13981 33943011 PMC8201545

[24] Hou X Gu YK Liu XW Fu JH Wang X Zhang LJ The impact of tumor cell differentiation on survival of patients with resectable esophageal squamous cell carcinomas. Ann Surg Oncol. (2015) 22(3):1008–14. 10.1245/s10434-014-4067-x 25201504

[25] Ma MQ Yu ZT Tang P Jiang HJ Zhao XJ Zhang JG Is tumor length a prognostic indicator for esophageal squamous cell carcinoma? A single larger study among Chinese patients. Int J Clin Exp Pathol. (2015) 8(5):5008–16. 10.1155/2021/8571438 26191194 PMC4503066

[26] Pasquer A Gronnier C Renaud F Duhamel A Théreaux J Carrere N Impact of adjuvant chemotherapy on patients with lymph node-positive esophageal cancer who are primarily treated with surgery. Ann Surg Oncol. (2015) 22(Suppl 3):S1340–9. 10.1245/s10434-015-4658-1 26065869

[27] Akiyama H Tsurumaru M Udagawa H Kajiyama Y. Radical lymph node dissection for cancer of the thoracic esophagus. Ann Surg. (1994) 220(3):364–72. 10.1097/00000658-199409000-00012 8092902 PMC1234394

[28] Baba M Aikou T Yoshinaka H Natsugoe S Fukumoto T Shimazu H Long-term results of subtotal esophagectomy with three-field lymphadenectomy for carcinoma of the thoracic esophagus. Ann Surg. (1994) 219(3):310–6. 10.1097/00000658-199403000-00012 8147613 PMC1243140

[29] Li J Qiu R Hu Y Wang Y Qi Z He M Postoperative adjuvant therapy for patients with pN+ esophageal squamous cell carcinoma. Biomed Res Int. (2021) 2021:8571438. 10.1155/2021/8571438 33553432 PMC7847342

[30] Wang Q Lang J Li T Peng L Dai W Jiang Y Postoperative adjuvant chemotherapy versus chemoradiotherapy for node-positive esophageal squamous cell carcinoma: a propensity score-matched analysis. Radiat Oncol. (2020) 15(1):119. 10.1186/s13014-020-01557-9 32448253 PMC7245784

[31] Ganti AK Williams CD Gajra A Kelley MJ. Effect of age on the efficacy of adjuvant chemotherapy for resected non-small cell lung cancer. Cancer. (2015) 121(15):2578–85. 10.1002/cncr.29360 25873330

[32] Khattak MA Townsend AR Beeke C Karapetis CS Luke C Padbury R Impact of age on choice of chemotherapy and outcome in advanced colorectal cancer. Eur J Cancer. (2012) 48(9):1293–8. 10.1016/j.ejca.2011.09.029 22119202

